# An Overview of Cell Membrane Perforation and Resealing Mechanisms for Localized Drug Delivery

**DOI:** 10.3390/pharmaceutics14040886

**Published:** 2022-04-18

**Authors:** Stephanie He, Davindra Singh, Brandon Helfield

**Affiliations:** 1 Department of Biology, Concordia University, Montreal, QC H4B 1R6, Canada; stephanie.he@concordia.ca (S.H.); davindra.singh@concordia.ca (D.S.); 2 Department of Physics, Concordia University, Montreal, QC H4B 1R6, Canada

**Keywords:** wound healing, plasma membrane repair, exocytosis, endocytosis, membrane permeability, cytoskeletal remodeling, pore, sonoporation

## Abstract

Localized and reversible plasma membrane disruption is a promising technique employed for the targeted deposition of exogenous therapeutic compounds for the treatment of disease. Indeed, the plasma membrane represents a significant barrier to successful delivery, and various physical methods using light, sound, and electrical energy have been developed to generate cell membrane perforations to circumvent this issue. To restore homeostasis and preserve viability, localized cellular repair mechanisms are subsequently triggered to initiate a rapid restoration of plasma membrane integrity. Here, we summarize the known emergency membrane repair responses, detailing the salient membrane sealing proteins as well as the underlying cytoskeletal remodeling that follows the physical induction of a localized plasma membrane pore, and we present an overview of potential modulation strategies that may improve targeted drug delivery approaches.

## 1. Introduction

Drug delivery techniques have revolutionized the field of precision medicine, helping to convert promising therapeutics into successful therapies [1]. The overall concept is to locally deliver high concentrations of therapeutics, either actively or passively, to the disease site and minimize off-target deposition. In so doing, the major limitations of systemic drug administration can be curtailed, including low solubility, poor biodistribution, unfavorable pharmacokinetics, and lack of selectivity [2]. Indeed, the major classes of therapeutic compounds, including small molecules, proteins and peptides, monoclonal antibodies, nucleic acids, and live cells, have all been incorporated into drug delivery systems and made significant contributions towards the treatment of disease [3]. Despite this exciting progress, there remain significant challenges in designing drug delivery tools, most notably in maintaining therapeutic stability, target specificity, and penetration of biological barriers (e.g., cell membranes) [4].

The current techniques for intracellular delivery can be broadly characterized into two sub-types: carrier-mediated delivery and membrane-permeating delivery [2,3,4,5]. Carrier-mediated approaches rely on biochemical constructs, including drug-loaded nanoparticles, viral vectors, and extracellular vesicles, to overcome some of the limitations of naked drug delivery [4]. Systems based on nanoparticle or microparticle constructs have allowed the deposition of otherwise low-solubility drugs, enabled the trafficking of small molecules to their site of action, and increased drug retention in tumour sites [6]. Environmental modifications, including the addition of cell-penetrating peptides, can aid in plasma membrane penetration and endosomal escape [3].

Membrane-permeating strategies are physical methods that use an external force to puncture the cell membrane and allow direct access to the intracellular space, thereby bypassing the need to overcome the plasma membrane barrier and escape from early endosomes. However, unlike carrier-based methods, the target cells must respond in a timely manner to repair the temporary damage sustained to the plasma membrane [7,8,9]. Individual eukaryotic cells can quickly repair their plasma membranes after injury through a sequential, highly localized process that restores internal homeostasis and prevents cell death [8,9,10]. The physical perforation methods currently used for drug delivery use external forces of different origins, including electric fields, ultrasound, and light, and thus are expected to result in characteristically different pore dynamics, including the spatiotemporal coordination of the key components involved in wound repair.

While aspects of cellular membrane pore repair mechanisms have been previously reviewed, the current manuscript aims to link the physical methods of membrane perforation with membrane repair biomechanics and to identify techniques that may be implemented for the development of improved drug delivery systems. We first provide a brief description of the main physical methods employed for local or targeted drug/gene delivery into cells and discuss what is known about the spatial–temporal behaviour of plasma membrane perforations using these modalities. Following this, we provide a description of the subcellular and molecular events that restore bilayer integrity, highlighting the protein families implicated in membrane repair and the cytoskeletal-based mechanisms involved in pore resealing. Finally, we offer a discussion on the interplay between the fundamental study of wound repair on an individual cell level with targeted drug/gene delivery paradigms.

## 2. Overview of Physical Plasma Membrane Permeation Techniques

The physical disruption of the plasma membrane results from the spatially and temporally regulated deposition of energy. The following section provides a brief overview of the more common approaches used to achieve this (Table 1).

### 2.1. Microinjection

Perhaps the most direct and established technique for membrane permeabilization is microinjection using a fine-tipped micropipette, typically characterized by an outer diameter on the order of 200–1000 nm. Used to create a single membrane pore on a single cell for therapeutic delivery, this approach requires a precision translational stage and a micro-injector performed under a high-gain objective microscope [11]. Since its original application over forty years ago, microinjection has been a reliable technique for delivering nucleic acid to the cell cytoplasm or directly into the nucleus, which bypasses cytoplasmic degradation. Microinjection is an extremely efficient method for a variety of payloads, irrespective of particle size and charge, including peptides, proteins, and oligonucleotides, and the exact number of DNA molecules can be precisely controlled. It is, however, a low-throughput technique that is best suited for specialty applications. Indeed, it is currently widely used to generate transgenic animals [12] through microinjection of a transgenic construct into the pronucleus of a fertilized egg (oocyte or zygote), including mice, pigs, goats, and cattle (e.g., [13,14]), and it is also used in forms of in vitro fertilization [15].

### 2.2. Sonoporation

One of the more recent techniques to increase plasma membrane permeability uses ultrasound energy. Biomedical ultrasound is widely employed as an imaging modality for anatomical assessment, as well as to provide information on blood flow characteristics. As an acoustic wave is transmitted into the body, reflections are generated at tissue interfaces and recorded to generate an image [16]. Ultrasound contrast agent, which consists of a solution of encapsulated bubbles typically between 1 and 10 µm in size, gives rise to strong scattered echoes from the vasculature in which they are confined—much stronger than red blood cells [17]. Contrast-enhanced ultrasound imaging is currently employed clinically in cardiology and radiology applications to improve the delineation of vessel lumen and to enable the visualization of the microcirculation [18]. Microbubbles vibrate within an ultrasound field, expanding and contracting about their resting size, and exhibit a rich variety of dynamic behaviours that are functions of the transmit conditions (acoustic frequency, peak-negative pressure, pulse duration, and duty cycle), the intrinsic bubble properties (size, shell characteristics, and constituents), and the local boundary conditions (vessel constraints) [19]. These behaviours range from stable and spherically symmetric vibrations to shape distortions, bubble fragmentation, and violent bubble collapse. It has been shown through numerical simulations and careful experimental investigations that microbubble oscillations create complex local fluid dynamic patterns; when situated adjacent to vessels, they can create local shear stresses that may ultimately modulate vasoactivity [20], vascular permeability [21], and local cell membrane perforation [22]. Indeed, a microbubble acts as a force actuator, focusing ultrasonic energy on the millimeter scale (typical wavelengths 0.75≤λ≤3 mm) to micro-manipulate neighboring plasma membranes [23] or generate an individual sub-micron- to micron-sized membrane perforation [24,25]—a process termed sonoporation. Under the assumption that blood is a Newtonian fluid, the shear stress τ due to a vibrating microbubble of size $R_{0}$ can be estimated as

(1) $$\tau \approx 2{\left(\mu \rho \right)}^{1/2}{\left(\pi f\right)}^{3/2}\left(\varepsilon R_{0}\right),$$

where μ is the fluid viscosity, ρ is the fluid density, f is the transit frequency, and ε is the maximum radial excursion of the bubble.

Among other design factors, recent work has demonstrated that microbubble proximity to the target cell is a key parameter in sonoporation efficiency, requiring distances on the order of a microbubble diameter or less between them [26]. Efforts to minimize microbubble–cell distances are currently being investigated, including the coupling of targeting ligands within the bubble encapsulation to promote site-specific microbubble accumulation (e.g., $\alpha_{V}\beta_{3}$ [27]), and novel ultrasound pulse sequences to initiate microbubble translation towards neighboring cells using acoustic radiation force. Investigations employing static techniques post-treatment, including scanning electron microscopy and atomic force microscopy, reveal sonoporation-induced pore diameters ranging from 10 to 1200 nm in diameter [28,29,30,31] and, depending on the acoustic conditions, broadly consistent with microscopy studies that infer these spatial scales from intracellular fluorescence tracer uptake dynamics interpreted via diffusion models [32]. Real-time microscopy approaches that directly observe and quantify membrane perforation during sonoporation events [24,33] have shown that these pores exhibit rapid opening timescales (<1 min) and longer resealing timescales (>1–10 min) and can resemble transmembrane apertures. Given the fundamental nature of sonoporation, that is, the generation of very-high-magnitude shear stress (~>kPa) acting on very short timescales (~µs), the spatial–temporal characteristics of microbubble-assisted membrane perforation and their relationship to different cell types are not well understood.

Since the discovery of the potential for microbubble-mediated therapeutic delivery in the 1990s [34], there have been many investigations into sonoporation efficiency within the fields of cardiovascular disease [35], brain disorders [36], cancer [37], and immunotherapy [38] that highlight the successful delivery of therapeutic macromolecules, plasmid DNA, mRNA, oligonucleotides, and associated viral vectors. Perhaps the simplest approach towards microbubble-mediated drug delivery is via a co-injection, whereby local therapeutic macromolecules migrate to the extravascular or intracellular space through sonoporation-derived perforations due to passive diffusion. Current pre-clinical and clinical trials using MR-guided microbubble-mediated blood–brain barrier disruption employ this technique for localized drug delivery [39]. Through advances in microbubble synthesis techniques, other platforms are being developed that incorporate therapeutic payloads into the bubble itself, including drug loading within the encapsulation material and strategies that attach payloads to the surface of the microbubble shell (e.g., electrostatic interactions [40] or nanoparticle linkage [41]). For gene delivery applications, these constructs have shown an increased resistance to nucleic acid degradation within blood serum [42] and thus exhibit a significantly longer half-life than otherwise unshielded gene approaches.

Given that microbubbles are currently clinically approved for ultrasound contrast imaging, sonoporation- and ultrasound-microbubble-assisted therapies present an inherently image-guided in vivo approach to targeted drug delivery. These therapies fit many requirements of an ideal gene delivery platform, such as minimal procedural invasiveness, limited off-target deposition due to tight acoustic focussing and biochemical ligands, ease of repeated treatments, and a good safety profile in preclinical studies (see Table 1). Additionally, current advances in device development have introduced techniques for passively detecting and quantifying regions of microbubble-treatment in real-time for the purposes of treatment monitoring and quality control [43].

### 2.3. Electroporation

Electroporation is a technique whereby cellular membranes exhibit increased permeability to macromolecules when exposed to an external electric field. While the mechanisms are not yet fully elucidated, it is generally accepted that nanopores are generated within the plasma membrane upon exposure to high-magnitude electric fields of a given duration. Under physiological conditions, a cell maintains a potential difference across its plasma membrane of approximately −50 to −80 mV, in which its intracellular contents maintain a slightly negative charge compared to the extracellular environment [44]. Under an applied external E-field $E_{ext}$, the induced transmembrane potential $\Delta \Psi_{m}$ across a cell membrane of effective radius $R_{c}$ is generally given by [45]:

(2) $${\Delta \Psi }_{m}=f_{s}E_{ext}R_{c}\cos\theta ,$$

where θ is the polar angle between the normal vector of the electric field and the site on the membrane at which $\Delta \Psi_{m}$ is evaluated and $f_{s}$ is a dimensionless term related to the electrical properties of the cytosol, plasma membrane, and the extracellular compartment, typically taken as $f_{s}\approx 1.5\;$for most mechanistic studies [46]. To achieve enhanced cell membrane permeability, a transmembrane potential threshold on the order of $\Delta \Psi_{m}\approx 1$ V is required, slightly dependent on cell type [46,47]. The generation and characterization of these nanopores are dependent on the pulse parameters, including pulse height, width, and duration. Electroporation typically generates many pores within the plasma membrane, with theoretical estimates of pore density on the order of $10^{9}$ pores/cm^2^ [48], the majority of which are < 1 nm in radius [49]. The kinetics of the transmembrane transport that is achieved with this approach can be approximated in five stages: (i) pore initiation (~0.1–1 µs); (ii) expansion (~ms), lasting as long as the pulse remains above the threshold value; (iii) stabilization (~ms), a stable decrease in permeability while the pulse is turned off; (iv) resealing (seconds to hours), the return to baseline permeability; and (v) the gradual cessation of residual memory effects (hours), which refers to the observation that cells, even after full membrane resealing, still exhibit alterations in their physiological processes before returning to their equilibrium state [50].

Similar to other types of physical permeation strategies, electroporation can be divided into two distinct types: reversible electroporation (RE), in which the nanopores are transient and the plasma membrane integrity is restored, or irreversible electroporation (IRE), in which the perforations do not reseal, resulting in cell death. Indeed, electroporation has been used successfully to introduce a variety of molecules into cells (e.g., [51,52]), including ions, drugs, RNA, micro-RNA, and DNA. While many studies have investigated the optimal parameters, a starting point for conditions that achieve cell permeation with high cell viability (i.e., RE) is applying eight square waves of 100 μs in duration at a frequency of 1 Hz and an amplitude of 1.2 kVcm^−1^—with the recognition that other factors, including cell size (Equation (1)), temperature, and the desired therapeutic agent, may play a role in these applied parameters [46]. Ex vivo applications typically involve blood cells treated outside the body and then reintroduced to provide therapeutic benefits. Electroporation has been employed on stem cells [53], to introduce chimeric-antigen receptor genes in T cells [54], and to modify red blood cells [55]. For in vivo applications of this technique, naked injection of the therapeutic into the target tissue is required prior to the application of the external E-field. The E-field is generated via electrodes placed in direct contact with the tissue, and therefore target regions are limited to those that both the therapeutic and the electrodes can access safely. This being said, in vivo electroporation has been demonstrated in the liver, bladder, brain, muscle, and skin (e.g., [56,57]).

It is important to note that recent works have investigated IRE as a primary, desired endpoint [44]. As a non-thermal tissue ablation modality capable of treating clinically sized volumes of tissue, under certain conditions, this approach allows for the preservation of collagenous and other protein/lipid-based structures, including the vasculature [58]. This relative advantage over other ablative approaches, as well as its relatively short treatment time requirement, has motived investigations into many soft-tissue cancer types (e.g., [59,60]), including pancreas, prostate, liver, lung, and brain—resulting in more than 50 clinical trials since its inception over a decade ago [44].

### 2.4. Photoporation

Photoporation, otherwise referred to as optoporation, is a technique in which highly focused light is the source of membrane perforation. In this technique, a laser beam is typically focused on a spot with a size on the order of 0.5–1 µm by a high numerical aperture microscope objective lens to the plasma membrane of a cultured monolayer. Photoporation has been demonstrated using continuous-wave light exposure, as well as pulsed laser modes, including pulse durations in the millisecond, nanosecond, and femtosecond timescales (e.g., [61]). Modifying the operating mode of the laser and its physical characteristics, such as wavelength and energy density, alter the physical and chemical mechanisms for induced cell membrane perforation [62]. Continuous-wave operation likely relies on heat deposition to induce membrane perforation and is often performed in the presence of an absorbing dye in the culture media. Although it causes perforation on a single cell level with high resulting cell viability, the pores generated by continuous-wave approaches are not as efficient as other modes [61]. Pulse laser sources with very high irradiances (e.g., $10^{10}-10^{12}$ Wcm^−2^) locally generate large E-fields ($10^{6}-10^{7}$ Vcm^−1^) compared to the average intramolecular Coulomb fields, resulting in the breakdown of target molecules in the focal region [63]. With slightly longer pulses in the nanosecond range, this can be accompanied by heating, bubble formation, and thermoelastic stress [64] that can expand the spatial scale of perforation to tens of microns [65]. Pore sizes generated by this technique range from ~10 to 1000 nm, depending on the parameters of the laser source [66]. Currently, the most widely adopted approach to optoporation is the use of femtosecond lasers (~<200 fp pulse durations), typically at 800nm [61]. Since the original demonstration over thirty years ago to deliver DNA into rat kidney cells [67], a wide array of membrane-impermeable substances have been delivered in this way with high cell viability, including dyes, nanoparticles, DNA, and mRNA (e.g., [68,69]). Additionally, photoporation can be achieved in combination with gold nanoparticles to increase the likelihood of perforation for a given set of input conditions (e.g., [70]), presumably due to the local amplification of the E-field [62]. Optoporation provides a means to robustly perforate cell membranes; however, it is mostly restricted to in vitro applications due to the low penetration depth in vivo and the requirement for complex optical setups.

## 3. Plasma Membrane Repair Mechanisms

**Figure 1:**
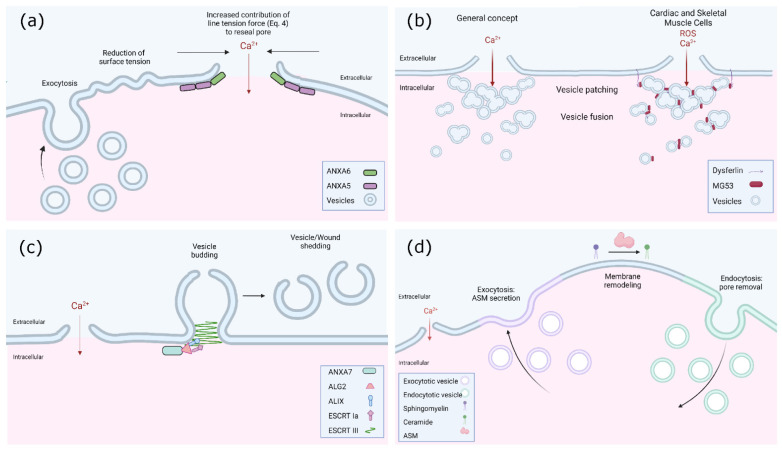
Summary of known plasma membrane resealing mechanisms. (**a**) The tension–reduction hypothesis; (**b**) the patch hypothesis; (**c**) the ESCRT mechanism; and (**d**) exocytosis/endocytosis. See text for details.

Pore generation within the plasma membrane launches an immediate cellular response to restore homeostasis and preserve cell viability. In this section, the known mechanisms of cell membrane repair are described (Figure 1) and summarized in Table 2.

### 3.1. Physical Intuition

As a first approach to understanding the dynamics of membrane pore resealing, it is perhaps instructive to consider the flux through a circular pore in the absence of any biological wound response. Assuming the cytoplasm is a simple fluid leaking out of a single pore of radius $r_{p}$, the flow per second normalized to initial cell volume $\tilde{Q}$ is given by the following relation [85]:

(3) $$\tilde{Q}=\left(\frac{\Delta P}{4\pi \eta_{0}}\right){\left(\frac{r_{p}}{R}\right)}^{3},$$

where ΔP is the pressure difference across the membrane, R is the effective cell radius, and $\eta_{0}$ is the cytosolic viscosity. Assuming ΔP≈ 10 Pa, an intracellular viscosity range from $\eta_{0}\approx$ 1 to 200 times that of pure water, and a pore size of 10% of the cell size, the leakage rate is on the order of 80%–4% of cell volume per second. From such a purely physical analysis, the necessity of viable cell membrane resealing occurring within seconds to minutes is clear. Membrane pore resealing times, quantified as a cessation of intracellular influx, have been reported over an array of input sources (e.g., ultrasound and microinjection) and across different cell types to range on this timescale [24,86,87,88,89].

### 3.2. Repair Triggers

It is well established that calcium ions are involved in a plethora of signaling pathways and cellular processes and, as such, intracellular Ca^2+^ concentration is well regulated. Due to the 10,000-fold gradient maintained across the plasma membrane [90], a localized breach of the plasma membrane results in an immediate calcium ion influx and is considered the universal trigger that launches the mechanisms of perforation repair [9]. Indeed, it presumably dictates the magnitude of plasma membrane repair as entry levels are approximately correlated to pore size, duration, and/or density, while an excessive level of intracellular Ca^2+^ is cytotoxic [8]. Previous works have demonstrated that membrane resealing timescales increase in low Ca^2+^ environments and fail to reseal in the complete absence of extracellular Ca^2+^ [8,91,92]. Calcium entry through membrane perforations selectively activates Ca^2+^-dependent cellular responses depending on both the local Ca^2+^ concentration and the relative affinity of Ca^2+^ binding proteins [93]. This enables cells to mount a spatially and temporally regulated response to steep Ca^2+^ influx [78]. The extracellular Ca^2+^ concentrations required for successful resealing have been reported in the µM to mM range [94], depending on the cell type and the wound generating mechanism, and tolerable increases in intracellular Ca^2+^ also vary between cell types. This highlights that tight control over Ca^2+^ dynamics is required to maintain cell viability following membrane perforation.

Oxidation at the plasma membrane is another trigger that initiates repair mechanisms in skeletal and cardiac cells [95]. Previous studies conducted on myotubes have elucidated the role of the protein Mitsugumin 53 (MG53) in muscle cell repair [96]. In the presence of a reducing agent in the extracellular environment, the accumulation of MG53 is hindered compared to the addition of an oxidizing agent, which increases the rate of MG53 accumulation at the site of cellular injury. MG53 also interacts with dysferlin to be translocated to the region where the concentration of free radicals is highest to seal the pore in a “patching” manner, typically within a minute after membrane injury [79]. The role of MG53 in membrane fusion, budding, and exocytosis is modulated by muscle-specific caveolin-3 (Cav3) for proper sarcolemma repair during muscle contractions and differentiation [80].

### 3.3. Plasma Membrane Repair Hypotheses

A breach of the plasma membrane disrupts the tension sustained by the lipid bilayer. Nanosized pores are frequently and transiently formed on cellular membranes as the cell naturally synthesizes organelles, moves, or contracts [97]. In these cases, the lipid bilayer can reseal these pores without requiring a cascade of proteins. Given this, there have been many experimental and theoretical investigations of wound healing on pure lipid vesicles, whereby the passive pore-opening and resealing dynamics are due to the force balance per unit length between two opposing forces:

(4) $$F=2\sigma \left(r_{p}\right)-\frac{\lambda }{r_{p}},$$

where $\sigma (r_{p})$ is the plasma membrane surface tension that acts to pull the pore of radius $r_{p}$ open, and λ is the line tension that is associated with the energy penalty of having exposed hydrophobic lipids along the pore perimeter and acts to close the pore. The relatively simplistic model given above predicts that pore size, opening, and resealing times are a function of lipid composition and cell viscosity [85,98].

Early experiments in sea urchin cells and mammalian cells revealed the localized fusion of intracellular vesicles with the plasma membrane, leading to two fundamental hypotheses for wound resealing [9]: (i) the ‘tension reduction’ hypothesis stipulates that the excessive membrane surface area delivered via the exocytosis of pre-existing intracellular vesicles serves to rapidly decrease the membrane surface tension to promote wound closure (see Equation (4)); and (ii) the fusion of pre-existing vesicles in the vicinity of the pore, including lysosomes, create a ‘patch’ that merges with the plasma membrane to seal the wound. Although the mechanisms underlying plasma membrane repair in mammalian cells are not fully elucidated and may differ between cell types and the source of the perforation, increasing evidence suggests that annexins are one of the first to be recruited to the wound site [74]. The annexin family, consisting of twelve proteins (A1–A11, A13), is made up of phospholipid-binding proteins that are triggered to migrate from the cytosol to the plasma membrane under local increases of Ca^2+^ concentration as early as 10–45 s post-perforation [99]. As each of these proteins exhibits its own Ca^2+^ sensitivity threshold, this family presents a broad Ca^2+^ sensing mechanism that responds dynamically during a wound repair event. There is evidence that annexins play a role in the immediate ‘patching’ of the perforation in an attempt to minimize cytosolic loss and intracellular Ca^2+^ increase. Annexin protein A5, when recruited to the perforation site, has been shown to form a 2D protein array to temporarily delay diffusion and limit wound expansion [100]. Annexin protein A4 binds to the plasma membrane adjacent to the opening and changes conformation to reshape the lipid bilayer, while annexin A6 constricts the wound to prevent it from expanding and to promote closure [73]. Synaptotagmin 7 (SYT7), a member of the synaptotagmin protein family, is a Ca^2+^-sensing protein present on the membrane of lysosomes that plays a critical role in lysosomal fusion and membrane patching, as its inhibition leads to impaired membrane repair [101]. Further, SYT7, among other members of its protein family, regulates the formation of soluble N-ethylmaleimide sensitive factor attachment protein receptor complexes (SNARE), which are a large group of small proteins that are key mediators of all intracellular membrane fusion events [102]. As opposed to SYT7 acting as a Ca^2+^-dependent modulator of membrane fusion, another Ca^2+^ sensor, ALG-2, modulates membrane fission machinery to mediate pore repair. ALG-2 recruits important membrane-trafficking scaffold proteins, including ALG-2 interacting protein X (ALIX), which then recruits components of the endosomal sorting complex required for transportation (ESCRT) machinery [8]. ESCRT-III, normally associated with its role in multivesicular body biogenesis, has been shown to translocate to the wound site through the recruitment of ALIX, ESCRT-1a, and ESCRT-II [74,82,103]. Indeed, under specific perforation conditions, a lack of ALG-2 or ALIX results in failed membrane repair [83]. The ESCRT machinery has, therefore, recently been suggested as a third alternative mechanism in addition to the patching and membrane tension reduction hypotheses, in which membrane lesions are actively removed through the formation of vesicles outwards from the damaged membrane—the shedding of membrane buds [82,104], see Figure 1c.

While lysosome fusion was originally only thought to contribute to membrane patching, recent observations of massive Ca^2+^-dependent endocytosis following lysosomal exocytosis [105] have revealed a fourth hypothesis for membrane pore repair: the ‘exocytosis/endocytosis’ pathway (Figure 1d). The fusion of lysosomes to the site of injury promotes the release of lysosomal enzyme acid sphingomyelinase (ASM), which remodels the outer leaflet of the plasma membrane. This novel form of endocytosis, which takes place within seconds of membrane injury, has been shown to be induced by the modification of plasma membrane lipid sphingomyelin into ceramide [106], resulting in the formation of large domains capable of inward budding [107].

It should be stated here that, although presented as separate mechanisms, it is possible that multiple approaches to plasma membrane repair occur simultaneously and synergistically. For example, ESCRT-III recruitment to the cell membrane has been observed when membrane tension is low [103], and lysosomal (and other intracellular vesicles) fusion can contribute to all pathways. The threshold perforation size for these mechanisms is not yet fully elucidated but likely plays a role in the extent to which one mechanism is favoured over others.

## 4. Cytoskeletal Remodeling during Perforation

In the context of physical permeation strategies, cell membrane perforation often results in a local disruption of the actin cortex. As a consequence, pore recovery depends on the spatial–temporal coordination between key protein families involved in membrane recovery, cytoskeletal architecture, and vesicle fusion. The following section outlines the salient proteins involved in these processes and is summarized in Table 3.

### 4.1. Initial Reaction: Deconstruction of Actin Network

Local Ca^2+^ influx due to membrane perforation activates cytoskeletal-remodeling proteins, such as INF2 and calpain protease, that allow for the disassembly of the local cortical actin [113]. This disassembly helps reduce membrane surface tension and improves access for intracellular vesicles to fuse with the plasma membrane [10]. Indeed, studies have shown that the presence of actin depolymerization agents, such as DNAse 1, acts to enhance the reparation of damaged cell membranes, while actin stabilizing agents result in a decrease in the resealing rate [114]. Further, this disassembly has been shown to occur for small and large membrane lesions [115] and is likely a requirement irrespective of perforation size. Similar to the actin network, the microtubule network also undergoes a brief period of local disassembly at the damaged site [116]. The perforation pathway triggers the recruitment of the microtubule-associated protein 1 (EB1), which both promotes the re-assembly of microtubules and facilitates the transport of lipids to the wound area [88]. Indeed, given that directional vesicle transport to the site of perforation is controlled by myosin and kinesin activity through the reorganized elongated microtubules [117], a rapid repair and remodeling of the breached cytoskeletal architecture is required for viable perforation repair.

### 4.2. Resealing and Remodeling: Actomyosin Contractile Ring

One such remodeling mechanism is the formation of an actomyosin ring that has been shown in multiple models, including *Xenopus* oocytes and *Drosophila* embryos [78]. For this process, two elements of the cytoskeleton are required: actin and myosin II. These are both recruited to the wound edge, assemble as contractile arrays surrounding the perforation, and continuously contract throughout the repair process. The spatial–temporal regulation of the actomyosin ring is regulated by Ca^2+^-dependent Rho GTPases, specifically Cdc42 and RhoA [78]. Indeed, the Rho family of GTPases [118] plays a major role in cytoskeletal regulation and, consequently, is involved in cell migration, adhesion, cytokinesis, and perforation repair. GTPases act as molecular switches that can modulate signal transduction pathways in response to a specific stimulus. Activated RhoA accumulates in a ring around the perforation area (i.e., a RhoA activity zone) and spatially overlaps with myosin II, while the concentric Cdc42 activity zone overlaps with the actin ring [119]. Myosin II is recruited by the RhoA activation of Rho-associated kinase (ROK) [110], and the Cdc42 activity zone is responsible for the recruitment and polymerization of branched actin filaments through downstream effectors, such as N-WASP and p21-activated kinases, that control the Arp2/3-dependent actin assembly [120]. Overall, the formation of these zones has been shown to occur within 30–45 s in *Xenopus* and *Drosophila* models [121] and has been shown to be active for the repair of wounds within the micrometer range [122]. Microtubules are also shown to be essential in the recruitment of Arp2/3 and myosin II and help focus the zone of actin and myosin II assembly at the wound edge [109]. Together, these proteins create an actomyosin cable around the wounded area that is coupled to the plasma membrane by junction proteins such as E-cadherin and B-catenin. In E-cadherin-deficient cell models, wound overexpansion and improperly formed actomyosin rings are observed, yet complete wound repair remains possible, suggesting that other proteins are involved in the tethering of the actomyosin ring to the plasma membrane [123]. The circular constriction shortens the actomyosin cable that pulls the membrane closer together to close the wound and is often referred to as the ‘purse-string’ mechanism. This mechanism is also seen in multicellular epithelial models, in which the Rho GTPase and ROK are essential for the assembly actomyosin ring intracellularly between the nearby cells [124]. This actomyosin ring is then anchored to the membrane through adheren and tight junctions, such as E-cadherin and ZO-1 [125], while constriction is initiated by the phosphorylation of myosin regulatory light chain (MLC) [126].

Though this repair model has been fully explored and described in embryonic mammalian models and multicellular epithelial models, the formation of the contractile ring is still yet to be seen in single somatic cells [127]. However, even though the ring itself is not seen, individual components are still shown to play a major role in cytoskeletal restructuring for membrane repair. Cdc42 and Rho, for example, are shown to be translocated from the cytosol to the membrane for cytoskeletal reorganization in shear-stress-damaged bovine aortic endothelial cells [128]. In laser-wounded skeletal muscle cells, RhoA activity is induced by Ca^2+^ influx [129], and F-actin accumulation at the wound site has been shown post-perforation in human endothelial cells [87], cancer cells [71], and muscle cells [111].

### 4.3. Resealing and Remodeling: S100A11-A2

In somatic cell models, another cytoskeleton remodeling mechanism has been demonstrated that utilizes the Ca^2+^ binding protein S100A11 and annexin A2. These proteins have been shown to be active in wounded mammalian cells, such as in cancer cells and vascular endothelial cells [71,77]. With the influx of extracellular calcium, the EF hand-type protein, S100A11, is activated and binds to F-actin along with annexin A2, which is also capable of Ca^2+^-dependent membrane binding [71]. The binding of these proteins to the cortical actin of the wounded membrane restricts the depolymerization of the F-actin while also promoting the buildup and increase in the polymerization of actin at the wounded zone [71]. The increase of cortical F-actin results in wound closure, and it is suggested to be related to the purse-string closure mechanism due to the buildup of cortical actin being analogous to the actin activity in wounded *Xenopus* oocytes [71]. Concurrently, annexin A1 accumulates on the damaged region of the membrane and labels it for excision [71]. In combination, the S100A11-A2 complex is able to bind to the membrane and pull it closer together, while annexin A1 removes damaged membranes. The necessity of this complex was shown in S100A11- and A2-depleted endothelial cells, in which pore resealing was either delayed or failed completely in both laser- and glass-beads-induced membrane damage models [130]. Overall, these components provide an alternate cytoskeletal remodeling mechanism for wounded membranes.

### 4.4. Resealing and Remodeling: Repair Cap

In muscle cells, another mechanism referred to as a “repair cap” is shown to occur post-membrane-wounding [111,131]. In this instance, an influx of Ca^2+^ results in the translocation of annexins towards the wounded membrane, particularly annexin A1, A2, A5, and A6; these accumulate and form a “cap” along the damaged region of the plasma membrane. This annexin-rich cap is supported both by a “shoulder” structure, shown to be essential for the repair cap formation, and consisting of proteins including dysferlin, EHD1/2, MG53, and BIN1 [8,111], and by F-actin recruitment to the non-annexin region below the cap. The formation of the repair cap is shown to be both Ca^2+^-and actin-dependent, demonstrating the necessity of cytoskeleton remodeling for this membrane repair mechanism [72,111].

### 4.5. Resealing and Remodeling: Exo/Endocytosis Events

All of the aforementioned plasma membrane repair pathways include steps that involve membrane fusion, exocytosis, or endocytosis, and in this manner the cytoskeleton plays a vital part in perforation repair (Table 3). All of the highly regulated steps of exocytosis, including the shuttling of vesicles to the peripheral membrane, vesicular fusion with the plasma membrane, and the release of vesicular content, are regulated by the actin cytoskeleton [112]. As exocytic vesicle size varies as a function of secretory cell type (~0.01–1 µm), so too does the timescale over which exocytosis occurs (~0.1–100 s), exhibiting an inverse relationship [112]. Vesicle transport along actin filaments requires actin-based motor proteins such as kinesin or members of the myosin V family [117]. While the molecular details differ between different vesicles, myosin Va is associated with several vesicles and has been shown to play a major role in this regard (e.g., endothelial cells [132] and neurons [133]). After membrane fusion by SNARE complexes [134], it has been suggested that the final expulsion of the vesicles utilizes an actin coat/ring at the base of the vesicle [135]. This mechanism is shown in laser-ablated HUVECs, in which Ca^2+^ influx results in the exocytosis of Weibel–Palade bodies, secretory organelles involved in the initiation of inflammation. In this model, Rho GTPases result in the recruitment of an actin ring at the base of the vesicle, while the contraction is enabled by myosin II-mediated constriction [136].

As stated above, the exocytosis-mediated release of ASM has been shown to result in a ceramide-coated membrane, ultimately triggering membrane invagination [137]. Indeed, studies have demonstrated that the transcriptional silencing of ASM inhibits membrane repair, while adding exogenous ASM can cause it to recover [106]. Recent work has suggested that the form of endocytosis that is stimulated by this mechanism is caveolin-dependent [138], implying that the usual role of the cytoskeleton in caveolin-mediated endocytosis is at play during perforation resealing under this pathway.

## 5. Future Perspectives

The investigation of individual cell wound repair mechanisms in the context of targeted drug and gene delivery can offer tremendous insight into the development of strategies designed to improve treatment outcomes through the modulation of perforation kinetics. This is especially of interest in optimizing in vivo techniques (e.g., sonoporation), whereby elucidating the mechanisms of perforation repair in human cells can aid in treatment design and planning. These strategies, whether physical or pharmacological, have applications both in targeted genetic delivery techniques (e.g., ischemia and cardiovascular disease) where preservation of cell viability is paramount and in cancer-related treatments where perhaps immediate and selective cancer cell death is the primary objective. Perhaps the most universal approach to altering the repair kinetics of individual cells is the administration of a Ca^2+^ chelator (e.g., BAPTA-AM) either during or immediately post-perforation [139]. As the key trigger and regulator of membrane repair, the local modulation of Ca^2+^ influx affects the spatial and temporal evolution of wound repair, potentially altering perforation size, repair timescale, and long-term cell viability via the activation/inhibition of Ca^2+^-dependent cellular responses [140]. The depletion of extracellular Ca^2+^ also induces the dissociation of intracellular junctions [141], including adherens and tight junctions, promoting paracellular drug transport. This may have a particular impact in aiding the targeted delivery of therapeutics to the brain via the reversible opening of the blood–brain barrier [142], an area in which microbubble-mediated focused ultrasound therapy, in particular, is rapidly progressing [36]. Further, there are novel advancements in the field of nanoparticle synthesis designed to modify the Ca^2+^ environment and affect local Ca^2+^ homeostasis. These techniques incorporate materials that are either Ca^2+^-coated [143] or Ca^2+^-binding [144] to influence the repair dynamics in wound healing applications.

The properties of plasma membranes, including lipid fluidity and phospholipid packing, play a role in their repair dynamics (e.g., the ‘tension-reduction’ hypothesis for perforation resealing). Indeed, lipid composition is regulated in response to pathological as well as pharmacological triggers. Membrane lipid alterations have been shown to be involved in many diseases, including cancer, atherosclerosis, and neurodegenerative diseases (e.g., [145]). One such example is the regulation of plasma-membrane-incorporated cholesterol, which may be dysfunctional in cancer cells [146] and result in variations in membrane fluidity and surface tension. Membrane cholesterol content is a key factor in modulating perforation repair dynamics, as has been demonstrated and physically modeled using giant unilamellar vesicles, resulting in shorter pore lifetimes with increasing cholesterol content [98]. Localized co-treatment with cholesterol depletion drugs, for example Filipin, ultimately decreases lipid raft number and membrane rigidity and may provide a means to ensure rapid resealing post-drug-delivery. Alternative lipid-altering strategies, such as incorporating pluronic polymers as part of the drug payload (e.g., Tween and polyethylene-glycol) [147], have the potential to preserve and amplify cell membrane recovery [148] and to facilitate intracellular drug transport [149]. Indeed, as polyethylene-glycol is a typical constituent of ultrasound-stimulated microbubbles used for sonoporation, microbubbles present a novel intrinsic vehicle for the local and targeted modulation of cell membrane viscoelasticity, as recently demonstrated [150]. It is well known that the composition of the outlet leaflet of the plasma membrane modifies its local surface tension and can trigger specific membrane repair mechanisms (e.g., the ‘exocytosis/endocytosis’ pathway), and this presents a unique strategy for tailoring drug/gene delivery treatment efficiency and timescales. Short-chain ceramides (with chain lengths four to eight carbons long) have been shown to readily incorporate into the outlet leaflet of the plasma membrane [151]. In contrast to their long-chained counterparts, short-chain ceramides are not able to form lipid rafts nor increase membrane rigidity [152]. Therefore, short-chain ceramides can be employed externally to increase membrane fluidity and have been used in conjunction with cancer therapeutics to improve intracellular drug permeability [153]. Indeed, the field of membrane lipid therapy, whereby the modulation of cell membrane composition can be achieved pharmacologically through membrane structure reorganization, regulation of enzymatic activity, and modulation of lipid-based gene expression [154], can be incorporated into targeted membrane-permeation strategies to tailor pore kinetics. In fact, cancer treatments based on membrane lipid therapy have been investigated in clinical trials of patients with advanced solid tumors (e.g., NCT02201823 and NCT01792310).

Aside from direct plasma membrane alteration, phospholipid-binding and cytoskeletal proteins offer promising targets for modulating perforation recovery dynamics, one of which is the annexin family of proteins. Previous studies have shown that treatment with annexin-1-derived peptides (e.g., Anx-12–26) elicits cyto-protective actions and shortens cardiomyocyte injury recovery time [155], facilitates wound healing in vivo [156], promotes epithelial repair [157], and can aid in the restoration of adheren junctions and the normalization of barrier integrity [158]. Recent studies have also demonstrated that treatment post-injury with recombinant annexin 6 enhances membrane repair capacity [159]. Further, annexin 5 has been investigated both as a diagnostic and as a therapeutic tool due to its strong binding affinity to phosphatidylserine, which results in an imaging surrogate for apoptosis in vivo and a potential anticoagulant, as it binds to activated platelets to prevent thrombin formation [160]. Additionally, annexin 5 has anti-inflammatory properties due to its potential to modulate nitric oxide signaling cascades, which has launched recent clinical trials investigating the delivery of recombinant human annexin 5 in patients with sepsis and COVID-19 (NCT04850339, NCT04748757). Tension development through the modulation of GTPases and cytoskeletal organization is also a promising approach to altering perforation kinetics [161]. The selective pharmacological inhibition of tension development, including the blocking of myosin II (blebbistatin), Rho-associated protein kinases (Y27632), and myosin light chain (ML-7), has been shown to result in a decrease in pore retraction time and prolongation of total perforation recovery, demonstrated in human dermal and lung microvascular endothelial cells [162], as well as neuroendocrine cells [163].

## 6. Conclusions

Cellular recovery from plasma membrane perforation is critical to ensure successful drug delivery using physical techniques. Indeed, fundamental molecular biology studies on individual cell membrane pore repair have brought tremendous insight into the arrangement of the key molecular actors at play. In coordination with salient Ca^2+^-dependent proteins, including the annexin family, various plasma resealing mechanisms have been put forth, including the ‘tension-reduction’, ‘patch’, ‘shedding’, and ‘exocytosis/endocytosis’ pathways. In conjunction with cytoskeletal reorganization involving actin and/or myosin and their roles in vesicular trafficking, these mechanisms likely work in concert to achieve rapid localized repair and control perforation dynamics. As such, physical membrane perforation techniques employed for targeted in vivo drug/gene delivery, including the use of microbubble-mediated ultrasound perforation, may benefit from co-administration with pharmacological agents to selectively modulate the spatio–temporal dynamics of membrane perforation to enhance therapeutic effectiveness.

## Figures and Tables

**Table 1:** A summary of the salient features of the main physical methods used to generate plasma membrane perforations.

Method	Pore Features	Relative Advantages	Relative Disadvantages
Microinjection	Single pore of similar size to the fine-tipped glass micropipette (~200–1000 nm).	Extremely efficient (~100%) Precise control over payload concentration	Very low throughput Highly technical Not applicable for in vivo drug delivery
Sonoporation	Pore radii ranging from sub-micron to ~10 µm. Single pore per bubble, with the possibility of multiple pores per cell.	In vivo translatability Drug/gene loaded constructs for added spatial targeting Image-guided Non-invasive	Highly dependent on ultrasound transmit and physical acoustic parameters
Electroporation	Pore radii generally < 1nm, with up to 10^9^ pores per cm^2^.	Very good efficiency Efficient for ex vivo applications	Semi-invasive procedure Limited in vivo applications Requires therapeutic co-injection
Photoporation	Pore radii ranging from ~10–2000 nm.	Very good efficiency, depending on the laser mode of operation	Low throughput Limited in vivo applications Requires therapeutic co-injection

**Table 2:** A summary of the key proteins involved in plasma membrane repair. See text for details.

Protein Family	Role	Ca^2+^ Binding	Estimated Pore Sizes in Which Proteins Have Been Observed	Suggested Plasma Membrane Repair Mechanism(s)
Annexins	Play a role in membrane patching, fusion, reshaping, reducing membrane tension, removing damaged membrane, limiting pore expansion	Yes	∼nm-μm [71,72,73]	Patch, Tension Reduction, Exocytosis/Endocytosis, Membrane Budding (A7 [74])
SNARE proteins	Mediates fusion of membranes	No	0.5–3 μm [75,76]	Patch, Tension Reduction, Exocytosis/Endocytosis
SYT7	Helps activate SNAREs	Yes		
S100A11	Implicated in membrane and cytoskeletal dynamics, interacts with A2	Yes	0.5–1.2 μm [71,77]	Tension Reduction [78]
Dysferlin	In muscle cells, accumulates at the site of membrane damage, interacts with some annexins, MG53, BIN1, EHD1/2	Yes	nm scale [79], μm scale [72]	Patch, Exocytosis/Endocytosis
MG53	In muscle cells, it is tethered to plasma membrane and intracellular vesicles and, upon ROS stimulus, oligomerizes and accumulates at wound sites	No	nm scale [79]	Patch [80], Tension Reduction [81]
ESCRT-III	Involved in membrane budding	No	<100 nm [82], >1 μm [83]	Membrane Budding
ESCRT-I	Recruits ESCRT-III	No		
ALIX	Recruits ESCRT machinery	Yes		
ALG-2	Recruits ALIX	Yes		
ASM	Outer plasma membrane remodeling to initiate inward vesicle budding. Converts sphingomyelin into ceramide	No	nm scale [84]	Exocytosis/Endocytosis

**Table 3:** A summary of the involvement of cytoskeletal remodeling post-pore formation. See text for details.

Cytoskeletal Remodeling	Main Proteins Involved	Estimated Pore Sizes in Which Proteins Have Been Observed
Contractile Ring [108]	Actin, myosin II, GTPases (Cdc42, Rho), Arp2/3,	∼μm scale [109,110]
S100A11-A2 [71]	S100A11, annexins A1, A2	0.5–1.2 μm [71,77]
Repair Cap [111]	Annexins A1, A2, A5 and A6, dysferlin, EHD1/2, MG53, BIN1	∼μm scale [72]
Exocytosis/Endocytosis (e.g., [112])	Myosin family, kinesin, actin, microtubules, GTPases, formins, SNARE complexes	

## Data Availability

Not applicable.
